# Neue Leitlinie für das Diabetesmanagement bei chronischer Nierenerkrankung

**DOI:** 10.1007/s00108-023-01485-2

**Published:** 2023-02-06

**Authors:** Christoph Wanner, Martin Busch

**Affiliations:** 1 Medizinische Klinik und Poliklinik I, Universitätsklinikum Würzburg, Oberdürrbacher Str. 6, 97080 Würzburg, Deutschland; 2 Klinik für Innere Medizin III, Universitätsklinikum Jena, Am Klinikum 1, 07747 Jena, Deutschland

**Keywords:** Natrium-Glukose-Kotransporter-2-Hemmer, Nichtsteroidale Mineralokortikoidrezeptorantagonisten, Glucagon-like-peptide-1-Rezeptor-Agonisten, Glykämische Kontrolle, Organ protection, Sodium-glucose transporter 2 inhibitors, Mineralocorticoid receptor antagonists, non-steroidal, Glucagon-like peptide‑1 receptor agonists, Glycemic control, Organ protection

## Abstract

Im Herbst 2022 wurde ein Update der Kidney Disease: Improving Global Outcomes (KDIGO) Clinical Practice Guideline zum Diabetesmanagement bei chronischer Nierenerkrankung („chronic kidney disease“ [CKD]) veröffentlicht. Im vorliegenden Beitrag werden die im Vergleich zu den Leitlinien von 2020 neuen Aspekte übersichtlich dargestellt und diskutiert. Neuerungen finden sich im Bereich der allgemeinen und allumfassenden Behandlung sowie hinsichtlich der blutzuckersenkenden und organprotektiven Therapien mit Natrium-Glukose-Kotransporter-2-Inhibitoren, nichtsteroidalen Mineralokortikoidrezeptorantagonisten und Glucagon-like-peptide-1-Rezeptor-Agonisten. Neu sind auch Top-10-Empfehlungen zum Diabetesmanagement bei CKD sowohl für Patient*innen als auch für Ärzt*innen. Die KDIGO-Leitlinien zum Diabetesmanagement bei CKD stellen den aktuellen evidenzbasierten Therapiestandard für Patienten mit Diabetes mellitus und CKD dar. Diesen gilt es nunmehr umzusetzen, um den betroffenen Patienten den Nutzen der Therapien zukommen lassen zu können und somit ihr Leben zu verbessern.

## Abstract (en)

In autumn 2022, an update of the Kidney Disease: Improving Global Outcomes (KDIGO) Clinical Practice Guideline on diabetes management in chronic kidney disease (CKD) was published. This article presents in a clear manner and discusses the new aspects compared to the 2020 guideline. Innovations are seen in the area of general and all-encompassing treatment as well as in relation to blood glucose-lowering and organ-protective treatments with sodium-glucose cotransporter 2 inhibitors, non-steroidal mineralocorticoid receptor antagonists, and glucagon-like peptide‑1 receptor agonists. A new feature is also the top 10 recommendations on diabetes management in CKD for both patients and physicians. The KDIGO guideline on diabetes management in CKD represents the current evidence-based standard of care for patients with diabetes mellitus and CKD. It is now important to implement the guideline in order to provide patients with the benefits of the treatments and thus improve their lives.

Im Oktober 2022 wurde ein Update der Kidney Disease: Improving Global Outcomes (KDIGO) Clinical Practice Guideline von 2020 zum Diabetesmanagement bei chronischer Nierenerkrankung („chronic kidney disease“ [CKD]) veröffentlicht [1, 2]. Zeitgleich wurde ein Consensus Report der KDIGO und American Diabetes Association (ADA) publiziert [3].

Ein neues Element der Leitlinien sind Top-10-Empfehlungen für Patient*innen und Ärzt*innen

Neue Aspekte im Vergleich zur Leitlinie von 2020 [4] ergaben sich in der allgemeinen und allumfassende Behandlung (umfassende medizinische Versorgung), aber auch bei den blutzuckersenkenden und organprotektiven Therapien mit Natrium-Glukose-Kotransporter-2-Inhibitoren (SGLT-2i), nichtsteroidalen Mineralokortikoidrezeptorantagonisten (nsMRA) und Glucagon-like-peptide-1-Rezeptor-Agonisten (GLP-1-RA). Die bisherigen Empfehlungen zu Blutzuckermonitoring, Zielwerten, Lebensstilinterventionen und der allgemeinen Herangehensweise blieben unberührt [4]. Neu sind auch Top-10-Empfehlungen sowohl für Patient*innen als auch für Ärzt*innen (https://www.kdigo.org; Tab. 1 und 2). Inhalte des vorliegenden Beitrags sind 3 neue Kernleitlinien, separate Praxisempfehlungen, die Top-10-Empfehlungen und zwei graphisch aufbereitete, anschauliche Abbildungen (Abb. 1 und 2).

**Table 1:** 

*1. Ganzheitlicher Ansatz:* Patient*innen mit Diabetes und CKD haben eine Multisystemerkrankung, die eine Behandlung mit grundlegenden Lebensstilinterventionen (gesunde Ernährung, Bewegung, Gewichtskontrolle, Nichtrauchen) sowie eine medikamentöse Therapie erfordert, die auch Nieren- und kardiovaskuläre Komplikationen vermeidet (Glukose, Lipide, Blutdruck; Abb. 2)
*2. Ernährung:* Patient*innen sollten eine ausgewogene, gesunde Ernährung mit einem hohen Anteil von Gemüse, Obst, Vollkornprodukten, Ballaststoffen, Hülsenfrüchten, pflanzlichen Proteinen, ungesättigten Fettsäuren und Nüssen zu sich nehmen; auch sollte der Anteil von verarbeitetem Fleisch, raffinierten Kohlenhydraten und gesüßten Getränken reduziert werden. Salz (weniger als 2 g/Tag) und Proteinzufuhr (0,8 g/kgKG pro Tag) sind in Übereinstimmung mit den Empfehlungen für die Allgemeinbevölkerung
*3. SGLT‑2i:* Eine SGLT-2i-Therapie sollte bei Patient*innen mit T2D, CKD und einer eGFR von > 20 ml/min pro 1,73 m^2^ begonnen werden und auch bei niedrigeren eGFR-Werten fortgesetzt werden. SGLT-2i reduzieren deutlich das Risiko eines Fortschreitens der CKD, einer Herzinsuffizienz und atherosklerotischer kardiovaskulärer Erkrankung, auch wenn die Blutglukosespiegel im Zielbereich liegen
*4. Metformin:* Bei T2D-Patienten mit CKD sollte Metformin bis zu einer eGFR von > 30 ml/min pro 1,73 m^2^ gegeben werden. Bei diesen Patienten ist Metformin sicher, effektiv und kostengünstig in der Kontrolle des Blutzuckers und der Reduktion diabetesbedingter Komplikationen
*5. Blutzuckermonitoring und Zielwerte:* Der HbA_1c_-Wert sollte regelmäßig bestimmt werden. Die Zuverlässigkeit der Messung nimmt mit fortschreitender CKD ab, vor allem bei Dialysepatient*innen. Die Ergebnisse sollten mit Vorsicht interpretiert werden. CGM oder SMBG sind auch von Nutzen, vor allem bei Hypoglykämierisiko. HbA1c-Zielwerte sollten in einem Bereich von < 6,5 % bis < 8 % individualisiert werden
*6. GLP-1-RA:* Für T2D-Patient*innen mit CKD, die trotz einer Metformin- und SGLT-2i-Therapie individualisierte Blutzuckerzielwerte nicht erreicht oder die Medikamente nicht vertragen haben, wird ein lang wirksamer GLP-1-RA als Teil der Behandlung empfohlen
*7. RAS-Hemmer:* Patient*innen mit T2D, mit oder ohne Hypertonie, sollten einen RAS-Hemmer (ACE-Hemmer oder ARB) erhalten, sobald eine UACR von > 30 mg/g persistiert. Der RAS-Hemmer sollte bis zur maximal empfohlenen oder tolerierten Dosis titriert werden. Serumkalium- und -kreatininkontrollen sollten erfolgen
*8. nsMRA:* nsMRA reduzieren das Risiko der CKD-Progression und das Auftreten kardiovaskulärer Ereignisse bei Patienten mit T2D und residualer Albuminurie. Sie werden bei einer UACR > 30 mg/g, einem normalen Serumkalium und weiteren Standardbehandlungen in der Therapie vorgeschlagen. Serumkalium- und -kreatininkontrollen sollten erfolgen
*9. Diabetesmanagement:* Bei der Betreuung dieser Patienten sollte ein teambasierter und integrierter Ansatz im Fokus regelmäßiger Evaluierungen stehen. Um das Risiko von Komplikationen zu verringern, sollte eine Kontrolle multipler Risikofaktoren erfolgen und eine strukturierte Ausbildung zum Selbstmanagement des Nierenfunktionsschutzes gegeben werden
*10. Empfehlungen für die Forschung:* Für ein optimiertes Management eines Diabetes bei Nierenversagen liegen wenig Daten vor, miteingeschlossen Dialyse und Transplantation. Dies sollte ein wichtiger Schwerpunkt für künftige klinische Studien sein

*ACE* „angiotensin-converting enzyme“, *ARB* Angiotensin-II-Rezeptor-Blocker, *CGM* kontinuierliche Glukosemessung, *CKD* „chronic kidney disease“ (chronische Nierenerkrankung), *eGFR* geschätzte glomeruläre Filtrationsrate, *GLP‑1-RA* Glucagon-like-peptide-1-Rezeptor-Agonist, *HbA*_*1c*_ Hämoglobin A_1c_, *nsMRA* nichtsteroidaler Mineralokortikoidrezeptorantagonist, *RAS* Renin-Angiotensin-System, *SGLT-2i* Natrium-Glukose-Kotransporter-2-Inhibitor, *SMBG* Selbstmessung der Blutglukose, *T2D* Diabetes mellitus Typ 2, *UACR* Albumin/Kreatinin-Verhältnis im Urin

**Table 2:** 

*1. Ganzheitlicher Ansatz:* Eine gesunde Ernährung, Bewegung, Rauchstopp sowie die Einstellung des Blutzuckers und der Blutfette unter Verwendung einer sinnvollen Medikation können das Risiko von Nierenversagen, Herzinfarkt, Schlaganfall und Herzinsuffizienz verringern (Abb. 2)
*2. Ernährung: *Ernähren Sie sich ausgewogen und gesund: Verwenden Sie Gemüse, Obst, Vollkornprodukte, Ballaststoffe, Hülsenfrüchte, pflanzliche Eiweißprodukte, ungesättigte Fettsäuren und Nüsse. Darüber hinaus sollten verarbeitete Fleischprodukte, einfache Kohlenhydrate wie Zucker oder Weißmehl und gesüßte Getränke vermieden werden. Die Salzaufnahme ist auf weniger als 2 g/Tag (gleichbedeutend mit 5 g Natriumchlorid) und die Proteinaufnahme auf 0,8 g/kgKG pro Tag zu begrenzen. Diese Werte sollten von einer Ernährungsberater*in begutachtet und dann regelmäßig kontrolliert werden
*3. SGLT-2i:* SGLT-2i wurden zur Senkung des Blutzuckerspiegels entwickelt, verringern aber auch das Risiko von Nierenversagen oder kardiovaskulärer Erkrankung. Sie können bei Menschen mit T2D und einer eGFR ≥ 20 ml/min pro 1,73 m^2^ eingesetzt werden
*4. Metformin: *Metformin sollte Bestandteil der initialen Therapie bei Patient*innen mit T2D sein, um den Blutzucker zu senken. Metformin sollte im Stadium 4 (eGFR < 30 ml/min pro 1,73 m^2^) abgesetzt werden
*5. Überwachung des Blutzuckerspiegels und der Zielwerte: *Der HbA_1c_-Wert sollte regelmäßig auch bei Patient*innen mit Diabetes und Nierenerkrankung gemessen werden. Die Messgenauigkeit von HbA_1c_ nimmt mit abnehmender Nierenfunktion ab. Vor allem ist sie nicht verlässlich bei Dialysepatienten und sollte mit Vorsicht interpretiert werden. CGM und SMBG sind sinnvoll, vor allem wenn das Hypoglykämierisiko erhöht ist. HbA1c-Zielwerte sollten an den Einzelnen angepasst werden und sich im Bereich von < 6,5 % bis < 8 % befinden. Immer sollte das Risiko einer Hyperglykämie berücksichtigt werden, vor allem bei fortgeschrittener Nierenerkrankung und in der Zusammenschau anderer blutzuckersenkender Medikamente
*6. GLP-1-RA:* Falls bei T2D, trotz Verwendung von Metformin und eines SGLT-2i, die Blutzuckerkontrolle nicht im Zielbereich liegt oder wenn diese Medikamente nicht vertragen werden, kann Ihnen Ihr Arzt einen lang wirksamen GLP-1-RA verschreiben. Dieser wirkt blutzuckersenkend und schützt auch das Herz
*7. RAS-Hemmer:* Ein ACE-Hemmer oder ein ARB – beides sind blutdrucksenkende Medikamente mit einem nierenschützenden Effekt – sollten bei T1D oder T2D eingenommen werden, wenn ein hoher Blutdruck vorliegt und/oder Eiweiß im Urin gemessen wurde. Das Eiweiß wird auch Albumin genannt. Die Nierenfunktion und der Kaliumspiegel im Blut sollten regelmäßig gemessen werden
*8. nsMRA:* nsMRA reduzieren das Risiko des Fortschreitens der chronischen Nierenerkrankung und das Auftreten kardiovaskulärer Ereignisse bei Menschen mit T2D und Albuminurie trotz Standardbehandlungen. Sie werden für die Therapie bei Patient*innen mit T2D und einer UACR > 30 mg/g bei normalem Blutkaliumspiegel in Gegenwart anderer Standardbehandlungen vorgeschlagen
*9. Herangehensweise an die Behandlung: *Es ist von großem Nutzen, wenn Sie Ihre Krankheit verstehen. Seien Sie ein aktiver Teil des Teams, das Ihren Diabetes und die Nierenerkrankung behandelt. Die Selbstkontrolle ist wichtig, und die Kontrolle der vielen Risikofaktoren wird dazu beitragen, die Nierenfunktion zu schützen und das Risiko diabetesbedingter Folgen zu vermindern
*10. Werden Sie ein selbstbestimmter Patient:* Patient*innen mit Diabetes fühlen sich mit den Anforderungen an die Lebensstilveränderungen of überfordert. Setzen Sie sich kleine, tägliche Ziele, die Ihnen helfen, die anstehenden Aufgaben zu bewältigen: Beispiele sind: (a) Gesund essen: Verringere die Portionsgröße beim Abendessen. (b) Bewegung: Gehe 10 min mehr jeden Tag. (c) Termine mit dem Gesundheitsteam: Schreiben Sie jede Woche eine Frage zu Ihrer Behandlung auf. (d) Medikamente: Verstehen Sie, wofür jedes einzunehmende Medikament notwendig ist und warum Sie es einnehmen. (e) Nebenwirkungen und Komplikationen: Kennen Sie Ihre Risikofaktoren und was gute Lebensstilmaßnahmen sind. Wie wirken diese auf mögliche Komplikationen des Diabetes?

*ACE* „angiotensin-converting enzyme“, *ARB* Angiotensin-II-Rezeptor-Blocker, *CGM* kontinuierliche Glukosemessung, *CKD* „chronic kidney disease“ (chronische Nierenerkrankung), *eGFR* geschätzte glomeruläre Filtrationsrate, *GLP-1-RA* Glucagon-like-peptide-1-Rezeptor-Agonist, *HbA*_*1c*_ Hämoglobin A_1c_, *nsMRA* nichtsteroidaler Mineralokortikoidrezeptorantagonist, *RAS* Renin-Angiotensin-System, *SGLT-2i* Natrium-Glukose-Kotransporter-2-Inhibitor, *SMBG* Selbstmessung der Blutglukose, *T1D* Diabetes mellitus Typ 1, *T2D* Diabetes mellitus Typ 2, *UACR* Albumin/Kreatinin-Verhältnis im Urin

**Figure 1:**
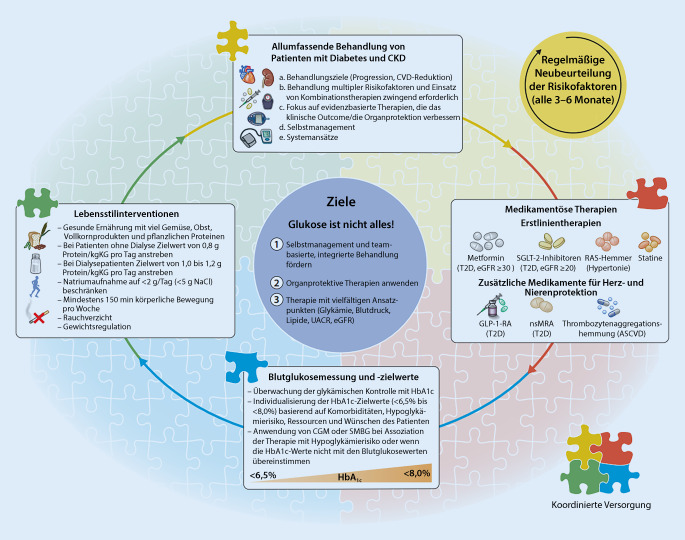

**Figure 2:**
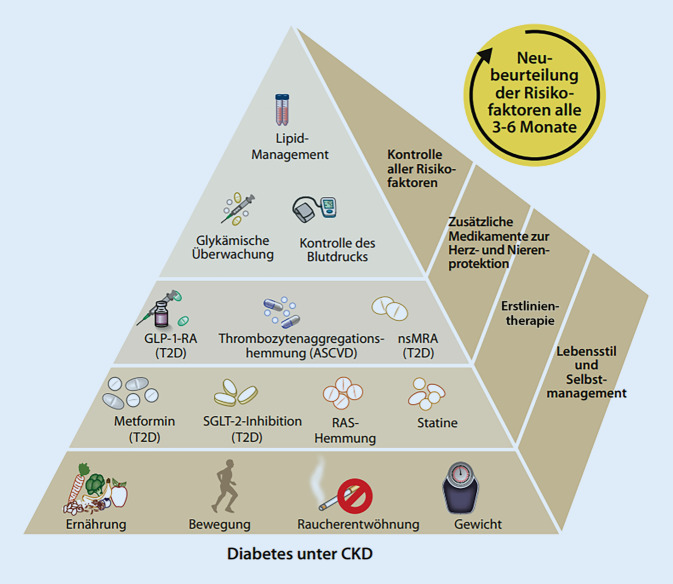

## Allumfassende Behandlung

Patient*innen mit Diabetes und CKD sollten mit einem ganzheitlichen Ansatz behandelt werden, um das Risiko der Nierenfunktionsverschlechterung und einer kardiovaskulären Erkrankung zu reduzieren (Abb. 1).

## Natrium-Glukose-Kotransporter-2-Inhibitoren

*Wir empfehlen, Patienten mit Diabetes mellitus Typ 2 (T2D), CKD und einer geschätzten glomerulären Filtrationsrate (eGFR) von ≥* *20* *ml/min pro 1,73* *m*^*2*^* mit einem SGLT-2i zu behandeln (1A).*

### Praktische Aspekte

1. Die Empfehlung zur SGLT-2i-Therapie dient der Nieren- und kardiovaskulären Protektion. Für SGLT-2i ist belegt, dass sie sicher sind und Vorteile für Patient*innen bieten, einschließlich derer ohne T2D. Falls Patient*innen bereits mit anderen blutzuckersenkenden Therapien behandelt werden, kann ein SGLT-2i zum bestehenden Therapieregime hinzugefügt werden.
2. Die Auswahl des SGLT-2i sollte Substanzen priorisieren, deren renaler oder kardiovaskulärer Nutzen belegt ist. Zudem sollte die eGFR einbezogen werden.
3. Es ist angemessen, SGLT-2i während prolongierter Nüchternphasen, chirurgischer Eingriffe oder kritischer Erkrankungen (sofern Patient*innen ein größeres Risiko einer Ketoazidose haben) zu pausieren.
4. Sofern eine Patient*in ein Risiko für eine Hypovolämie aufweist, sollte vor Beginn einer Therapie mit einem SGLT-2i die Dosisreduktion eines Thiazid- oder Schleifendiuretikums erwogen werden. Patienten sollten über Symptome eines Volumenmangels oder niedrigen Blutdrucks aufgeklärt werden, und der Volumenstatus sollte nach Therapiebeginn beobachtet werden.
5. Zu Beginn einer Therapie mit einem SGLT-2i kann ein reversibler Abfall der eGFR auftreten. Dies ist im Allgemeinen keine Indikation, die Therapie zu unterbrechen.
6. Sobald eine SGLT-2i-Therapie begonnen wurde, ist es sinnvoll, die Therapie auch bei einem Abfall der eGFR auf < 20 ml/min pro 1,73 m^2^ fortzusetzen, es sei denn, sie wird nicht toleriert oder eine Nierenersatztherapie wird eingeleitet.
7. SGLT-2i wurden bei Patienten nach Nierentransplantation, die von SGLT-2i profitieren könnten, nicht ausreichend untersucht. Patienten nach Nierentransplantation sind immunsupprimiert und mit einem potenziell erhöhten Infektionsrisiko behaftet, weshalb sich die Empfehlung zum Gebrauch von SGLT-2i nicht auf Nierentransplantatempfänger bezieht (siehe Empfehlung 1.3.1).

## Nichtsteroidale Mineralokortikoidrezeptorantagonisten

Wir schlagen für Patient*innen mit T2D, die trotz Therapie mit einer maximal tolerierten RAS-Hemmer-Dosis (RAS* Renin-Angiotensin-System) eine eGFR ≥* *25* *ml/min pro 1,73* *m*^*2*^*, ein normales Serumkalium und eine Albuminurie (≥* *30* *mg/g bzw. 3* *mg/mmol) aufweisen, einen nsMRA, mit belegtem Nieren- und kardiovaskulärem Nutzen, vor (2A).*

### Praktische Aspekte

1. Nichtsteroidale MRA sind am besten für Patient*innen mit T2D geeignet, die ein hohes Risiko einer CKD-Progression oder kardiovaskulärer Ereignisse aufweisen, was sich in einer persistierenden Albuminurie trotz anderer Standardtherapien zeigt.
2. Ein nsMRA kann der Therapie mit RAS-Hemmer und SGLT-2i für die Behandlung des T2D und der CKD hinzugefügt werden.
3. Um das Risiko einer Hyperkaliämie abzumildern, sollten Patient*innen mit einer anhaltend normalen Serumkaliumkonzentration ausgewählt werden und die Serumkaliumspiegel sollten nach einem Therapiebeginn mit nsMRA anhaltend kontrolliert werden.
4. Die Auswahl eines nsMRA sollte Substanzen priorisieren, die einen belegten renalen oder kardiovaskulären Nutzen haben.
5. Steroidale MRA sollten für die Behandlung einer Herzinsuffizienz, eines Hyperaldosteronismus oder einer therapierefraktären arteriellen Hypertonie eingesetzt werden, können jedoch eine Hyperkaliämie oder einen reversiblen Abfall der glomerulären Filtrationsrate (GFR) bewirken, insbesondere bei Patient*innen mit einer niedrigen GFR.

## Glucagon-like-peptide-1-Rezeptor-Agonisten

Wir empfehlen für Patient*innen mit T2D und CKD, die trotz Behandlung mit Metformin und SGLT-2i noch nicht das individualisierte Blutzuckerziel erreicht haben oder die nicht in der Lage sind, diese Therapie zu nutzen, einen lang wirksamen GLP-1-RA (1B).

### Praktische Aspekte

1. Die Auswahl des GLP-1-RA sollte Substanzen mit dokumentiertem kardiovaskulärem Nutzen bevorzugen.
2. Um gastrointestinale Nebenwirkungen zu minimieren, sollte mit einer niedrigen Dosis des GLP-1-RA begonnen werden und die Dosis langsam gesteigert werden.
3. GLP-1-RA sollten nicht in Kombination mit Dipeptidylpeptidase-4(DPP-4)-Hemmern verwendet werden.
4. Das Hypoglykämierisiko ist unter der Monotherapie mit GLP-1-RA in der Regel gering. Das Risiko ist erhöht, sofern GLP-1-RA zusammen mit anderen Medikamenten, wie Sulfonylharnstoffen oder Insulin, verwendet werden. Die Dosis von Sulfonylharnstoffen und/oder Insulin muss eventuell reduziert werden.
5. GLP-1-RA sollten bevorzugt bei Patient*innen mit Adipositas, T2D und CKD verwendet werden, um eine gewollte Gewichtsabnahme herbeizuführen.

## Diskussion

Die aktualisierte KDIGO-Leitlinie zum Diabetesmanagement bei CKD von Herbst 2022 bietet einen praktischen und evidenzbasierten Behandlungsansatz, der von Patient*innen und Behandler*innen begrüßt werden dürfte. Neue Erfassungen belegen, dass im Jahr 2021 bereits ein halbe Milliarde Menschen weltweit an Diabetes erkrankt waren. Die Vorhersagen bis 2040 erfüllen mit Sorge. Im Allgemeinen entwickeln 4 von 10 Personen mit Diabetes eine Nierenbeteiligung [1], potenziell mit Progression der Nierenerkrankung. Daraus ergibt sich die Empfehlung, bevorzugt den Erhalt der Nierenfunktion anzustreben, statt sie mittels Dialyse oder Transplantation zu ersetzen [1, 2]. Des Weiteren kann die Belastung durch kardiovaskuläre Erkrankungen verringert werden. SGLT-2i, nsMRA und GLP-1-RA eröffnen eine Perspektive, diese Ziele zu erreichen, um somit das Leben vieler Betroffener zu verbessern und Millionen Leben zu retten.

Ein Ziel ist jetzt die Implementierung der Leitlinie auf breiter Ebene

Dies bedarf jedoch des breiten und rechtzeitigen Einsatzes dieser Medikamente [2] und somit der Umsetzung der existierenden Evidenz in die tägliche Praxis. Die Kosten der neuen Therapien wurden von den Kostenträgern verhandelt und die Therapien erhielten aufgrund ihres besonderen Nutzens einen besonderen Stellenwert. SGLT-2i wurden in spezieller Indikation als Praxisbesonderheit anerkannt, und die Kostenersparnis durch Verhinderung einer Dialyse ist belegt. Das weitere Ziel wäre jetzt die Implementierung der Leitlinie auf breiter Ebene, aber auch das Screening auf Nierenerkrankungen. Letztere Empfehlung folgt aus einer gewissen Freude bzw. Erleichterung darüber, dass die Medizin 2023 den Betroffenen ein Angebot zur Beherrschung der Erkrankung unterbreiten kann. Unter Miteinbezug von Patient*innen besteht nun die besondere Hoffnung, diese wertvollen Therapien rechtzeitig und interdisziplinär implementieren zu können.

Um eine Inkonsistenz verschiedener Empfehlungen zu vermeiden, kooperiert KDIGO mit der ADA. Ganz aktuell ist auch der Consensus Report von KDIGO und ADA erschienen [3], der eine breite Übereinstimmung evidenzbasierter Empfehlungen beider Fachgesellschaften darstellt. „Die Wissenschaft ist global, aber umgesetzt wird sie lokal“ („Science is global, but implementation is local“) ist eine viel zitierte Erkenntnis aus KDIGO. Alle KDIGO-Leitlinien wurden bisher von der Deutschen Gesellschaft für Nephrologie geprüft und unterstützend gebilligt. Die KDIGO-Leitlinie zum Diabetesmanagement bei CKD stellen den aktuellen evidenzbasierten Therapiestandard für Patienten mit Diabetes mellitus und CKD dar. Diesen gilt es nunmehr umzusetzen, um den betroffenen Patienten den Nutzen der Therapien zukommen zu lassen und somit ihr Leben zu verbessern [2].

## Fazit für die Praxis

- Die aktualisierte Leitlinie von Kidney Disease: Improving Global Outcomes (KDIGO) zum Diabetesmanagement bei chronischer Nierenerkrankung bietet einen praktischen und evidenzbasierten Behandlungsansatz, der von Patient*innen und Behandler*innen begrüßt werden dürfte.
- Neue Aspekte im Vergleich zu den Leitlinien von 2020 finden sich in der allgemeinen und allumfassende Behandlung, aber auch in der Therapie mit Natrium-Glukose-Kotransporter-2-Inhibitoren, nichtsteroidalen Mineralokortikoidrezeptorantagonisten und Glucagon-like-peptide-1-Rezeptor-Agonisten.
- Die bisherigen Empfehlungen zu Blutzuckermonitoring, Zielwerten, Lebensstilinterventionen und der allgemeinen Herangehensweise blieben unberührt.
- Es gilt nun, die Neuerungen umzusetzen, damit betroffene Patienten vom neuen Therapiestandard profitieren können.
